# An Energy-Efficient Strategy for Accurate Distance Estimation in Wireless Sensor Networks

**DOI:** 10.3390/s121115438

**Published:** 2012-11-09

**Authors:** Paula Tarrío, Ana M. Bernardos, José R. Casar

**Affiliations:** Data Processing and Simulation Group, Universidad Politécnica de Madrid, ETSI. Telecomunicación, Avda. Complutense 30, 28040, Madrid, Spain; E-Mails: abernardos@grpss.ssr.upm.es (A.M.B.); jramon@grpss.ssr.upm.es (J.R.C.)

**Keywords:** energy consumption, distance estimation, sensor networks

## Abstract

In line with recent research efforts made to conceive energy saving protocols and algorithms and power sensitive network architectures, in this paper we propose a transmission strategy to minimize the energy consumption in a sensor network when using a localization technique based on the measurement of the strength (RSS) or the time of arrival (TOA) of the received signal. In particular, we find the transmission power and the packet transmission rate that jointly minimize the total consumed energy, while ensuring at the same time a desired accuracy in the RSS or TOA measurements. We also propose some corrections to these theoretical results to take into account the effects of shadowing and packet loss in the propagation channel. The proposed strategy is shown to be effective in realistic scenarios providing energy savings with respect to other transmission strategies, and also guaranteeing a given accuracy in the distance estimations, which will serve to guarantee a desired accuracy in the localization result.

## 1. Introduction

Due to environmental concerns and to the limited lifetime of energy resources, an intensive research activity is being carried out in energy-efficient protocols and algorithms for wireless networks, trying to reduce the energy consumption of the different network activities.

One of the activities that should be optimized is the localization of the nodes, as many applications rely on the knowledge of the position of the user or the surrounding objects to provide useful information and services. Furthermore, knowing the position of the nodes can be used to optimize other network aspects, such as routing [1,2] or in-network data compression [3,4], leading to energy savings.

The localization of the nodes in wireless networks is usually based on radio frequency techniques [5], which consist in measuring certain parameters of the radio signal that one node receives from other nodes, for example, the angle of arrival (AOA), the time of arrival (TOA) or the received signal strength (RSS). From these measurements, the position of the wireless nodes can be inferred using a triangulation algorithm [6] or by comparing the measurement with others previously saved in a database [7,8].

The research in localization for wireless sensor and ad hoc networks is very active, and a number of works have considered the energy consumption as a factor in the design of localization techniques and algorithms. This is a critical issue, since wireless devices have limited energy resources (batteries), which should be efficiently managed in order to extend the life of the network as much as possible [9]. Consequently, practical and effective solutions are needed to perform the localization in an energy-efficient way.

Approaches that include the use of dedicated hardware specially designed for this purpose [10] cannot be used with commercial off-the-shelf systems. On the other hand, given that the radio system is the most energy demanding part of a wireless node [11,12], whereas the other elements (processor, memory, *etc.*) consume significantly less energy, solutions that focus on reducing the computational complexity of the localization algorithms [13,14] will not contribute to significantly reduce the global energy consumption. Therefore, to effectively reduce the amount of energy that is consumed during the localization, the aim should be put on reducing the amount of time that the nodes are communicating. In other words, the transmitted/received signals that are used to measure the parameter in which the localization is based (e.g., RSS, TOA) should be kept to the minimum.

However, energy consumption cannot be considered independently. The accuracy of the localization should be also taken into account, as in general, we could probably allow higher energy consumption if more accurate localization result is needed. In contrast to other works that focus on this trade-off by modifying the frequency of the localization to trade higher energy consumption for better localization accuracy (in terms of update rate) [15–18], we propose to trade the accuracy of the RSS or TOA measurements for energy consumption. Our approach can be applied regardless of the localization update frequency, thus it complements the existing work and further reduces the energy consumption. In particular, we propose and evaluate a strategy that minimizes the total consumed energy during the localization process, while ensuring a certain desired accuracy in distance estimations. Although we illustrate the results of the analysis for a particular channel and communications model, the method is directly extensible to other models. Our work includes an exhaustive analysis based on simulations, which shows that not only the energy consumption is reduced with respect to other techniques (leading to energy savings of around 50% in some cases), but also that the accuracy of the localization is improved and assured to maintain a desired value. The main contributions of this paper are: (1) we propose a strategy to minimize energy consumption during the localization process that assures a given localization accuracy and is based on local information at each node; (2) we include two effective modifications of the above theoretical strategy in order to consider the effects of the signal propagation in real environments.

The structure of the paper is as follows. Section 2 reviews some related work regarding the consumption-accuracy trade-off in localization systems. Section 3 defines the application scenario and the communication models that have been selected for our analysis. Section 4 analyzes the energy consumption of the localization procedure and proposes a transmission strategy, based on local information at each node, which minimizes the total power consumption of the network and at the same time achieves a given accuracy in the distance estimations. Some modifications to this theoretical strategy are also proposed to achieve the desired performance under real propagation conditions. The performance of the proposed strategy is evaluated in Section 5 and compared with other strategies through a set of simulations based on experimental measurements. Finally, Section 6 extracts some conclusions and proposes some future work.

## 2. Related Work

The trade-off between the energy consumption and the accuracy of localization algorithms was first addressed in [19], which proposed a localization algorithm in which each node can choose between different methods to measure the distances to their neighbors, each one with different accuracy and different energy consumption. This algorithm enables to know the amount of energy needed to obtain a given accuracy, or the accuracy that is achieved with a given energy consumption. As mentioned in Section 1, in wireless ad hoc and sensor networks distances are usually estimated from some parameter of the radio signal, as the RSS or the TOA. In practice, in a given network, only one of these parameters is used to estimate the distances between the nodes, so in order to reduce energy consumption, it would be valuable to find a transmission strategy that minimizes the energy consumption for a given accuracy and for a given distance estimation technique.

This idea is followed by some object-tracking systems, as [15–18], where the frequency of the localization is modified to trade higher energy consumption for better localization accuracy. Their general mechanisms consist in detecting or predicting the mobility pattern of the tracked target, and then dynamically adjusting the localization frequency. These methods are based on the assumption that the localization accuracy deteriorates with the target's mobility level due to a delay error produced by the movement of the object between the instant in which the RSS or TOA is measured and the instant in which the position is calculated. However, they do not consider that for a given localization frequency, the accuracy of the RSS or TOA measurements can also be traded for energy consumption.

In our previous work [20], we propose a communication scheme for RSS-based localization that minimizes the energy that is consumed during the measurement of the RSS while maintaining a given accuracy in this measurement. In [21] we extend this idea to TOA-based localization. The sensitivity of this strategy to errors in the distance estimation is evaluated in [22]. This paper builds on the strategy proposed in these previous works in order to gain a deeper insight into the energy consumption-localization accuracy trade-off. In particular, w.r.t. our previous work, we now provide a theoretical framework, showing that if all the involved parameters are expressed as a function of the signal to noise ratio, an optimal solution can be found in closed form. The advantages of this new formulation are twofold: (i) we capture the rationale of energy consumption by identifying its fundamental elements and their dependencies, and (ii) we achieve a considerable reduction in the computational complexity, as the closed-form solution avoids using a full search in the space of tunable parameters. Furthermore, we also extend previous work to take into account the effects of real propagation conditions, in order that the final strategy achieves the desired accuracy in the distance estimation even in real environments with shadowing and packet losses.

## 3. Problem Statement and Assumptions for the Localization Scheme

We consider here a typical scenario of a wireless multi-hop network composed of both mobile nodes and nodes that are deployed at fixed positions. In this scenario, the position estimation of the mobile nodes should be updated frequently enough so that the nodes can be tracked as they move. Hence, each localization of these nodes should be performed within a given period *T*_0_, which depends on the mobility pattern of each node. In this work, we assume that *T*_0_ is known. In fact, there are several methods available in the literature (e.g., [15–18]) to compute it in real time.

As explained in Section 1, the position of a node in a wireless network is usually computed from the measurements of a parameter of the radio signal (RSS, TOA, *etc.*). The accuracy of the localization will therefore depend on the accuracy of these measurements.

The measured radio parameters are random variables, so their uncertainty can be reduced, in general, by averaging a number of measurements. In particular, both TOA and RSS measurements are usually described as random Gaussian variables [23], so if *n* measurements are averaged, the standard deviation can be reduced by a factor of $\sqrt{n}$, provided the measurements are independent.

Let us suppose that a node will average the RSS or TOA of all packets received during a period *T*_0_, which depends on how often the localization of the node must be performed, with the objective of achieving a given accuracy in the distance estimations. The number of received packets (*n*) during this localization period will determine the accuracy of the RSS or TOA measurements, and therefore, the accuracy of the distance estimation. Due to the nature of the radio link, the receiver node will not receive all the packets that are transmitted by the transmitter node. The probability of receiving a packet in a certain link, that is, the packet reception probability (*PRP*) or packet reception rate, is a function of the signal to noise ratio (*SNR*), (see Section 3.1). Therefore, in order to receive *n* packets during a time interval *T*_0_, packets can be sent either with a high transmission power (so that the *SNR* at the receiver is high and very few transmitted packets are lost) at a rate of *n*/*T*_0_, or with a lower transmission power (some packets will be lost) but at a higher rate (*k*/*T*_0_, with *k* > *n* being the number of transmitted packets during the time interval *T*_0_).

The goal is to optimize the transmission strategy at each node with the aim of obtaining a given accuracy in the RSS or TOA measurements, while minimizing the energy consumption in the global network. As the radio system is the most energy demanding part of a node (see Section 1) we will only take into account the energy consumption of the radio system. The transmission strategy will be defined by two parameters: the transmission power (*P_TX_*) and the time between transmitted packets (*T*), that is, the inverse of the packet transmission rate (*k*/*T*_0_).

We will assume that all nodes in the network are synchronized; hence, one-way TOA measurements can be performed in the network. In addition, given that nodes are synchronized, they can turn off their radio when they are not transmitting or expecting to receive a packet from another node, so idle listening is avoided and the energy consumption of the radio system is only due to transmitting and receiving packets.

In order to evaluate the energy consumption during the localization procedure, some assumptions about the receiver and radio channel behavior must be done. In the following, we propose to use simple models of the receiver and the channel.

### 3.1. PRP-SNR Relation

The probability of a successful reception of a packet is the probability of successfully receiving all its bits, assuming that there is no error correction. It is well known that the probability of bit error depends on the encoding and modulation of the radio signal, thus, the packet reception probability will also depend on them. For example, for QPSK and BPSK modulation schemes, the packet reception probability in presence of additive white Gaussian noise and in absence of interferers is given by [24]:

(1) $$\text{PRP}={\left(1-\text{BER}\right)}^{8l}={\left(1-Q\left(\sqrt{2\frac{E_{b}}{N_{0}}}\right)\right)}^{8l}={\left(1-Q\left(\sqrt{2\text{snr}\cdot \frac{W}{v_{b}}}\right)\right)}^{8l}$$

where *l* is the length of the packet (in bytes), *BER* is the bit error rate, *E_b_/N*_0_ is the signal to noise ratio per bit, *snr* is the signal to noise ratio (in natural units), *W* is the channel bandwidth, *v_b_* is the bit rate and the function *Q*(*x*) is the tail probability of the standard normal distribution.

More complete models have been proposed [25] to take into account complex phenomena such as asymmetry, interference, different quality of receivers and transmitters, correlations between reception rate of links, *etc.* However, this simpler model has been selected for this work because it enables a closed-form mathematical formulation and also fits quite well to experimental data (see Section 5.1).

### 3.2. Path Loss Model

On the other hand, a model of the channel that relates the transmission power to the received power is needed, as it will provide a relation between transmission power *P_TX_* and *SNR* at the receiver for a certain distance between them. Many channel models have been proposed for outdoor and indoor environments [26] (e.g., Nakagami fading model, Rayleigh fading, Ricean fading, *etc.*) but one of the most popular, due to its simplicity, is the log-normal shadowing path loss model [27], which establishes the following relation between the received power (*P_RX_*) and the distance (*d*) between transmitter and receiver:

(2) $$P_{\text{RX}}(\text{dBm})=p_{\text{TX}}(\text{dBm})+A-10\eta \log\frac{d}{d_{0}}+𝒱$$

where *P_TX_* is the transmission power, *A* is a constant term, *η* is the path loss exponent, and 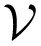 ∼ 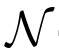
(0, *σ*^2^) is a zero-mean Gaussian random variable with standard deviation *σ*. The constant term *A* depends on the transmitter's and receiver's antenna gains and on the power loss for a reference distance *d*_0_, and has to be experimentally determined. On the other hand, the path loss exponent *η* typically ranges between 2 and 4 depending on the environment, and it has to be experimentally determined too. The random variable 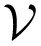 models the effects of shadowing and considers the randomness across an ensemble of many deployment environments. As the experiments in [28] indicate, if the environment is static and the transmitter and receiver stay at fixed positions, the standard deviation of the measured received power is lower than *σ*, whereas if a node moves or the environment changes (due to people movement or long lags of time) the standard deviation of the measured received power is *σ*. For this reason, if a channel model like Equation (2) is used to estimate distances, averaging a number of received signal strength measurements in a static situation does not significantly reduce the estimation errors, whereas averaging measurements taken at slightly different positions or in a changing environment is more effective to reduce estimation errors [29].

As previously said, this channel model provides a relation between the transmitted power (*P_TX_*), the *SNR* at the receiver and the distance (*d*) between transmitter and receiver, *i.e.*,:

(3) $$\text{SNR}(dB)=P_{\text{TX}}(\text{dBm})+A-10\eta \log\frac{d}{d_{0}}-N_{\text{RX}}(\text{dBm})+𝒱$$

where *SNR*, which is a random variable, is the signal to noise ratio at the receiver expressed in decibels and *N_RX_* is the noise level at the receiver.

## 4. A Transmission Strategy for Minimizing Energy Consumption

As explained in Section 3, our goal is to optimize the transmission strategy for the localization process with the aim of obtaining certain accuracy in the distance estimations while minimizing the energy consumption. In this section, we first include a theoretical analysis of the energy consumption during the localization procedure in terms of the transmission strategy parameters (transmission power and packet transmission rate) and the channel behavior (characterized through the *PRP*) in order to obtain general expressions valid for any wireless network. Some results are also presented for the particular models described in Section 3 and a minimum consumption transmission strategy for the localization process that guarantees a given accuracy in the distance estimations is proposed. Then, two modifications to this ideal strategy are introduced in order to make it practical and effective in real applications by counteracting the random effects of radio propagation.

### 4.1. Energy Consumption Analysis

Let us consider a certain link of a network like the one described in Section 3, consisting of a transmitter node, which will periodically send packets, and a receiver node, which will measure the RSS or the TOA of all received packets. The receiver will average all the measurements (*n*) obtained during the localization period *T*_0_, so that the standard deviation of the measurements can be reduced by a factor of $\sqrt{n}$. Therefore, a desired accuracy in the measurement of the RSS or the TOA imposes a minimum number of received packets *n_obj_*.

On the other hand, when *k* packets are transmitted, the average number of packets that are finally received is:

(4) $$n=k\cdot \text{PRP}=\frac{T_{0}}{T}\cdot \text{PRP}$$

where *T* is the time interval between consecutive transmitted packets. Consequently, a desired accuracy in the RSS or the TOA imposes a relationship between *T* and *PRP*, given *T*_0_. As shown in Section 3.1, the *PRP* depends on the *snr* at the receiver. As a result, a desired accuracy imposes a relationship between *snr* and *T*. Among all *snr-T* pairs that ensure the required number of received packets, we want to find the one with the lowest energy consumption.

The consumed energy at transmission during *T*_0_ is given by:

(5) $$E_{\text{TX}}=k\cdot E_{p}=k\cdot p_{\text{tx}}\cdot T_{p}=\frac{n\cdot p_{\text{tx}}\cdot T_{p}}{\text{PRP}}$$

where *k* is the number of transmitted packets during the period *T*_0_, *E_p_* is the consumed energy when transmitting a single packet, *p_tx_* is the transmission power (in natural units), *T_p_* is the time it takes to send a packet and *n* is the average number of received packets. Since the localization procedure is periodic with period *T*_0_, this consumed energy is an appropriate parameter to analyze the energy efficiency.

If we assume that the receiver is awake and listening to possible receptions only at given periods (previously negotiated with the transmitter), the consumed energy at reception during *T*_0_ is given by:

(6) $$E_{\text{RX}}=C_{\text{rx}}\cdot T_{r}$$

where *T_r_* is the reception period and *C_rx_* is the consumption when the node is in receiving mode. *E_RX_* is constant, as *C_rx_* is a constant value that depends on the hardware characteristics. Therefore, in order to minimize the total energy consumption, we will only need to minimize the energy consumption during transmission.

Using Equations (1) and (3), *E_TX_* can be expressed as a function of the signal to noise ratio at the receiver:

(7) $$E_{\text{TX}}=\frac{n\;T_{p}\;v_{b}\;d^{\eta }\;n_{\text{rx}}}{2\;Wa}\cdot \frac{x^{2}}{{\left(1-Q(x)\right)}^{8l}}\cdot \chi$$

where *n_rx_* and *a* are *N_RX_* and *A* expressed in natural units, *χ* is a log-normal random variable with parameters *μ_χ_* = 0 and *σ_χ_* = *σ* · ln 10/10, and *x* is given by:

(8) $$x=\sqrt{2\text{snr}\frac{W}{v_{b}}}$$

where *snr* is the mean value of the *SNR* expressed in natural units.

It can be obviously noticed that, for a given distance, the greater the required accuracy (described by *n*), the more energy will be consumed. On the other hand, as the distance between transmitter and receiver increases, the consumed energy increases as well, for a given desired accuracy. Finally, note that *E_TX_* is a random variable composed of a constant multiplied by a log-normal random variable, therefore, it is itself another log-normal random variable, with parameters $\mu_{E}=\ln\left(\frac{n\;T_{p}\;v_{b}\;d^{\eta }\;n_{\text{rx}}}{2\;Wa}\frac{x^{2}}{{\left(1-Q(x)\right)}^{8l}}\right)$, *σ_E_* = *σ_χ_* and mean value given by:

(9) $${\bar{E}}_{\text{TX}}=\frac{n\;T_{p}\;v_{b}\;d^{\eta }\;n_{\text{rx}}}{2\;Wa}\cdot \frac{x^{2}}{{\left(1-Q(x)\right)}^{8l}}\cdot e^{\frac{1}{2}{\left(\frac{\sigma \cdot \ln10}{10}\right)}^{2}}$$

Deriving Equation (9) with respect to the *snr* at the receiver, it can be obtained that the value of *x* (or *snr*) for which the average energy consumption reaches a minimum (x*) satisfies the following expression:

(10) $$\frac{x^{*}e^{-x^{*2}/2}}{\left(1-Q(x^{*})\right)}=\frac{\sqrt{2\pi }}{4l}$$

In conclusion, if we manage to achieve the snr* corresponding to this x*, the energy consumption will be minimized.

**Figure 1.**
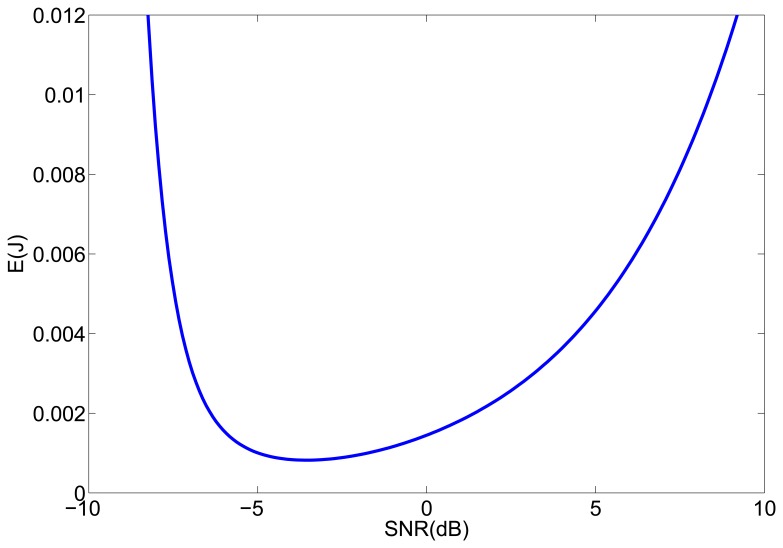
Energy consumption (in joules) as a function of the *SNR* at the receiver.

To provide some illustrative results, Figure 1 shows the energy consumption as a function of the *SNR* for the parameters of our sensor network, which performs a localization once a second, that is, *T*_0_ = 1 s, and uses 8-bytes packets, with *T_p_* = 256 *μ*s. The estimated values of the channel parameters are *η* = 2.5 and *A* = −80 dBm and the noise level at the receiver is *N_RX_* = −87 dBm. The energy consumption was calculated for *n* = 5 and *d* = 2 m. We can see in Figure 1 that if the optimum *SNR* value is not achievable, it would be better to use a higher transmission power, as the increase of power consumption with the *SNR* is slower for higher values of *SNR*.

On the other hand, note that the desired accuracy imposed a relationship between *snr* and *T*, thus, there is an optimum value of *T* corresponding to snr*, which can be calculated from Equations (1) and (4):

(11) $$T^{*}=\frac{T_{0}}{n}\cdot {\left(1-Q\left(\sqrt{2{\text{snr}}^{*}\cdot \frac{W}{v_{b}}}\right)\right)}^{8l}$$

In conclusion, among the pairs *snr*-*T* that satisfy a desired accuracy, there is a pair snr*-T* for which the energy consumption is minimum. For that reason, in order to measure the RSS or the TOA with certain accuracy, the strategy that we propose consists in transmitting packets with a packet rate of 1/T* and such a transmission power *P_TX_* that the *snr* at the receiver is equal to snr*. As the *snr* at the receiver is proportional to the transmission power for a given distance d between transmitter and receiver (see Section 3.2), in order to calculate the optimum value of *P_TX_*, an estimate of the distance between transmitter and receiver is needed. In practice, this distance estimation can be obtained from the RSS or TOA measurements of the previous localization period.

Note (from Equation (10)) that the optimum value x* only depends on the packet length, therefore, the optimum value of *snr* at the receiver to achieve the lowest energy consumption does not depend on the accuracy we want to obtain or on the propagation characteristics, it only depends on the packet length and on the *W*/*v_b_* ratio, *i.e.*, its value is constant for a given network. This is an interesting result from a practical point of view, as it implies that the value of snr* does not need to be calculated in real time, which would be costly from a computational point of view, as it involves solving expression (10) iteratively. Therefore, the proposed strategy only involves very simple operations to calculate the optimum values of T* and $P_{\text{TX}}^{*}$ from the known value of snr* and, yet, guarantees the desired accuracy in the measurements while achieving the minimum energy consumption. This results in a very useful and attractive technique for battery-powered wireless devices that are deployed to perform the localization of people or objects. Although we have derived here the particular expressions for the specific case of our channel and receiver models, the strategy is directly extendable to other models.

Let us also remark that the value of the localization period *T*_0_ does not have any influence on the applicability of our strategy as long as it is big enough to send the required number of packets, which is usually the case in practice. Furthermore, the value of *T*_0_ does not need to be constant in time. In fact, it can be optimized to adapt to the mobility characteristics of the mobile node [15–18]. The combination of the proposed optimization with the optimization of the localization period may lead to further energy savings.

Note that as we have assumed that there is a synchronization schedule known by all the nodes, the localization messages should be sent within the assigned periods. In principle, this does not affect the localization accuracy or the proposed strategy, as long as the required number of packets can be transmitted.

Finally, we would like to point out that in practice, the synchronization of the network may not be perfect. In this case, the nodes should be listening to possible transmissions also during the idle periods in order not to lose packets, so the energy consumption in reception mode would increase. The proposed strategy is not directly affected, as it optimizes the energy consumption in transmission mode, but the energy savings, relative to the total energy consumption, would not be as high as in the ideal synchronization case. Another effect of an imperfect synchronization is that TOA measurements will not be so accurate, so its standard deviation will increase and the distance estimation errors will be higher. This does not affect the performance of the proposed strategy, as it was formulated in terms of the desired number of packets. But clearly, the desired number of packets to achieve a certain accuracy will increase with respect to the ideal case (see Equation (25) in Appendix A: if *σ_t_* increases, *n* has to increase to achieve the same *σ*), and the energy consumption will increase accordingly.

### 4.2. Practical Considerations

In a real environment, the random nature of the propagation channel will have some effects on the proposed strategy that should be overcome. In particular, both the transmission power and the packet transmission rate need to be modified in order to maintain the desired accuracy when real propagation conditions are considered. In the rest of this section, we explain how and why these two parameters of the transmission strategy have to be corrected.

On one hand, the proposed strategy relies on the calculation of the transmission power that is necessary to reach the receiver with the optimum *snr*. As commented previously, an estimation of the distance between the nodes is necessary for this calculation. In real deployments, the distance estimations calculated from TOA or RSS measurements are affected by errors (see Appendix A and B), so the transmission power that is used to reach the receiver with the desired *snr* may be different from the optimum (see Section 4.3 for an analysis of how this fact affects the consumed energy). For example, in the case of RSS-based localization, in which distances are estimated from the received signal strength of the *received* packets, distances are usually underestimated. The reason is that the packets with low *snr* suffer from a higher loss probability and, thus, it is more probable to estimate the distance from higher-power packets, which will yield shorter distance estimations. If the transmission power corresponding to this underestimated distances is used, the packets will arrive at the receiver with a *snr* lower than snr*, and consequently, there will be more packet losses than desired. In order to avoid this effect, the transmission power has to be corrected.

According to Equation (3), the *SNR* (in decibels) of the packets that arrive at the receiver follows a normal distribution with mean $\bar{\text{SNR}}=P_{\text{TX}}+A-10\eta \log d/d_{0}-N_{\text{RX}}$ and standard deviation *σ*. Therefore, in natural units, the *snr* of these packets will be a log-normal random variable with parameters $\mu_{\text{snr}}=\bar{\text{SNR}}\cdot \ln10/10=\ln10^{\bar{\text{SNR}}/10}$ and *σ_snr_* = *σ* · ln 10/10. Among all the packets that reach the receiver, some are successfully received but some are not and the probability of a successful reception depends on the value of the *snr*, according to Equation (1). Thus, the probability density function of the *snr* given that the packet has been received can be expressed by:

(12) $$\begin{array}{cc} f_{\text{snr}/\text{rx}}(\text{snr}) & =\frac{f_{\text{snr}}(\text{snr})\cdot \text{PRP}(\text{snr})}{\int_{-\infty }^{+\infty }f_{\text{snr}}(\text{snr})\cdot \text{PRP}(\text{snr})\cdot d\text{snr}}\\ & =\frac{\frac{1}{\text{snr}\cdot \sigma_{\text{snr}}\sqrt{2\pi }}\cdot e^{-\frac{{\left(\ln\text{snr}-\mu_{\text{snr}}\right)}^{2}}{2\sigma_{\text{snr}}^{2}}}\cdot {\left(1-Q\left(\sqrt{2\text{snr}\cdot \frac{W}{v_{b}}}\right)\right)}^{8l}}{\int_{-\infty }^{+\infty }\frac{1}{\text{snr}\cdot \sigma_{\text{snr}}\sqrt{2\pi }}\cdot e^{-\frac{{\left(\ln\text{snr}-\mu_{\text{snr}}\right)}^{2}}{2\sigma_{\text{snr}}^{2}}}\cdot {\left(1-Q\left(\sqrt{2\text{snr}\cdot \frac{W}{v_{b}}}\right)\right)}^{8l}d\text{snr}} \end{array}$$

If one of the received packets is used to estimate the distance, the difference between the transmission power necessary to reach the receiver with the desired snr* calculated from it (*P̃_TX_*) and the optimum transmission power (given by the true distance) $\left(P_{\text{TX}}^{*}\right)$ can be expressed as:

(13) $$P_{\text{TX}}^{*}-{\tilde{P}}_{\text{TX}}=\text{SNR}-{\text{SNR}}^{*}$$

where *SNR* refers here to the *SNR* of the successfully received packet, whose probability density function can be calculated from Equation (12), knowing that *SNR* = 10 · log *snr*:

(14) $$f_{\text{SNR}/\text{rx}}(\text{SNR})=\frac{f_{\text{SNR}/\text{rx}}(\text{SNR})}{\frac{\partial \text{SNR}}{\partial \text{snr}}}=\frac{\ln10}{10}\cdot 10^{\text{SNR}/10}\cdot f_{\text{snr}/\text{rx}}(\text{SNR})$$

**Figure 2.**
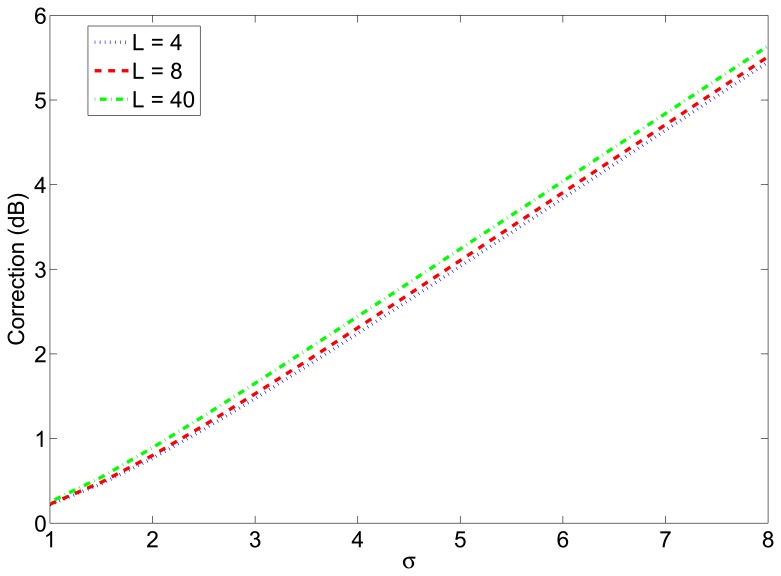
Correction margin for the transmission power calculated from estimated distances in RSS-based localization.

As a consequence, the estimated transmission power has to be corrected by adding a margin equal to the mean value of Equation (13). In other words, this margin must be calculated as the difference between the mean value of the *SNR* of the successfully received packets (which can be calculated numerically using Equation (14)) and SNR*. Figure 2 shows the value of this margin as a function of the standard deviation of the shadowing for different packet lengths. This margin was seen to be independent of the value of *W*/*v_b_*, and nearly independent of the packet length, as it can be seen in the figure.

**Figure 3.**
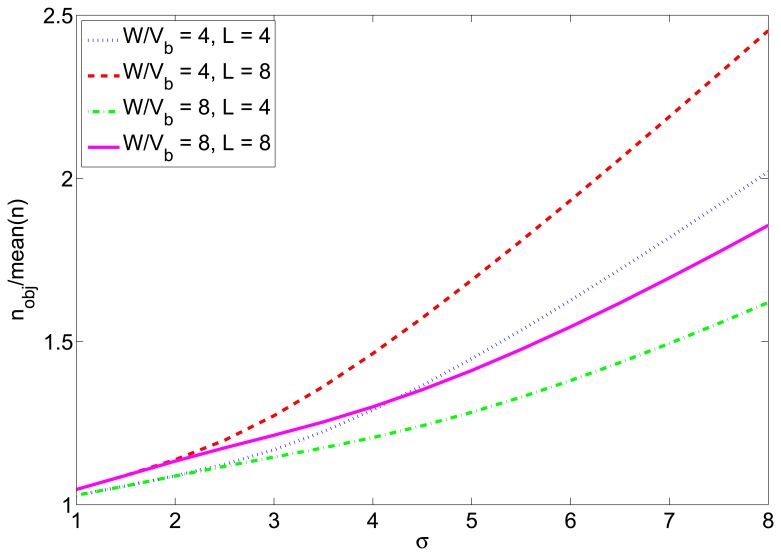
Ratio between the desired number of received packets and the mean value of *n*.

On the other hand, even when the optimum transmission power is used, the final *snr* at the receiver may not be the desired one, as the propagation is again affected by random shadowing. In fact, the *snr* at the receiver will be a log-normal random variable and, therefore, the *PRP* and *n* will also be random variables and their probability density functions can be calculated easily according to Equations (1) and (4). In particular, the probability density function of the *PRP* can be calculated as:

(15) $$f_{\text{PRP}}=\frac{f_{\text{snr}}}{\frac{\partial \text{PRP}}{\partial \text{snr}}}=\frac{\sqrt{2\pi }v_{b}}{8lW}\cdot \frac{x(1-Q(x))}{\text{PRP}\cdot e^{x^{2}/2}}\cdot \frac{1}{\text{snr}\cdot \sigma_{\text{snr}}\sqrt{2\pi }}\cdot e^{-\frac{{\left(\ln\text{snr}-\mu_{\text{snr}}\right)}^{2}}{2\sigma_{\text{snr}}^{2}}}$$

where $\mu_{\text{SNR}}=\ln10^{\bar{\text{SNR}}/10}=\ln{\text{snr}}^{*}$, *σ_snr_* = *σ* · ln 10/10, $x=\sqrt{2\text{snr}\cdot W/v_{b}}$ and *snr* = *v_b_*/2*W* · (*Q*^−1^ (1 − *PRP*^1/^*^8l^*)). Thus, knowing that *n* = *k* · *PRP*, the probability density function of *n* can be calculated as:

(16) $$f_{n}(n)=k\cdot f_{\text{PRP}}(n)=\frac{n_{\text{obj}}}{{\text{PRP}}^{*}}\cdot f_{\text{PRP}}(n)$$

where *n_obj_* is the desired number of received packets and PRP* is the value of the packet reception probability corresponding to the optimum *snr*: ${\text{PRP}}^{*}={\left(1-Q\left(\sqrt{2{\text{snr}}^{*}\cdot \frac{W}{v_{b}}}\right)\right)}^{8l}$. The mean value of *n* can be then calculated numerically, resulting that this value is lower than the desired one *n_obj_*. The difference between the two depends on l and *W*/*v_b_*: the mean value of *n* is lower when the packet is longer or when *W*/*v_b_* is higher. Figure 3 shows the relation between these two values for different values of l and *W*/*vb* for the case of RSS-based localization as a function of the standard deviation of the *SNR* distribution. This factor must be used to correct the number of transmitted packets, in order to counteract the effects of the propagation in real deployments. Thus, the estimated value of T* calculated from Equation (11) must be divided by this factor.

Although the energy consumption will increase when these two corrections are included in the transmission strategy, they are necessary to achieve the desired number of received packets in real environments and, therefore, to achieve the desired accuracy; otherwise, the accuracy would not be guaranteed. Nevertheless, the final energy consumption is very small and still entails significant savings with respect to other transmission strategies.

### 4.3. Sensitivity Analysis

It is interesting to analyze how much energy will be consumed if the best transmission strategy is not perfectly achieved, due to an error in the estimated transmission power. As it has been mentioned above, the error in the estimated transmission power is due to an error in the distance estimation, which depends itself on the RSS or TOA measurement errors and on the number of packets that are averaged at the receiver. In this section, we first evaluate how the energy consumption is affected by the errors in the estimated transmission power and then we study how the distance estimation errors propagate to errors in the estimated transmission power, and therefore affect the energy consumption.

Let us suppose a transmitter node X that has to send packets to another node Y, so that Y receives in average *n_obj_* packets during the period *T*_0_. As the optimum *snr* at receiver node Y is known, node X can calculate the optimum value of *T* (T*) that satisfies the desired accuracy using Equation (11). On the other hand, the optimum transmission power $P_{\text{TX}}^{*}$ can be obtained from Equation (3), assuming that the distance between transmitter node X and receiver node Y is known. An error in the distance estimation will produce an error in the estimated optimum transmission power, which will lead to the use of a transmission power $P_{\text{TX}}^{\prime }$ different from the optimum $P_{\text{TX}}^{*}$. If the estimated transmission power is greater than $P_{\text{TX}}^{*}$, the energy consumption will increase with respect to the minimum *E_min_* and the number of received packets will increase as well with respect to *n_obj_*, although it is not necessary. If the estimated transmission power is lower than $P_{\text{TX}}^{*}$, the energy consumption will diminish, but the number of received packets will not be sufficient.

**Figure 4.**
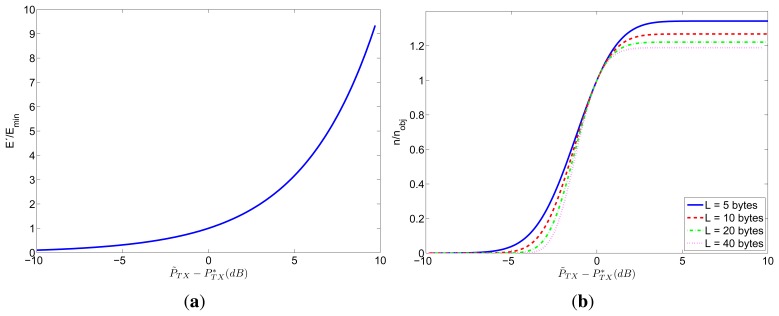
Effect of the error in the transmission power on the energy consumption and on the number of received packets.

Figure 4 shows how the errors in the transmission power affect the consumed energy and the number of received packets. In particular, Figure 4(a) represents the ratio between the energy consumption *E*′ corresponding to the estimated transmission power $P_{\text{TX}}^{\prime }$ and *E_min_* (corresponding to the optimum transmission power $P_{\text{TX}}^{*}$) as a function of the error in the transmission power $\left(P_{\text{TX}}^{\prime }-P_{\text{TX}}^{*}\right)$. This relation is valid for any value of *n_obj_*, for any *W*/*v_b_* ratio for any packet length l and for any distance between transmitter and receiver. On the other hand, Figure 4(b) shows the relation between the number of received packets *n* corresponding to the estimated transmission power $P_{\text{TX}}^{\prime }$ and the desired number of received packets *n_obj_* (corresponding to the optimum transmission power $P_{\text{TX}}^{*}$) as a function of the error in the transmission power. This relation is valid for any value of *n_obj_*, for any *W*/*v_b_* ratio and for any distance between transmitter and receiver, but depends on the packet length *l*.

As shown in Figure 4(a), the consumed energy *E*′ will be greater than the minimum value (*E_min_*) if the transmission power is overestimated. Furthermore, the relation between the error in the transmission power $\left({\tilde{P}}_{\text{TX}}-P_{\text{TX}}^{*}\right)$ and the corresponding energy consumption is exponential. For example, if the transmission power is overestimated in 1 dB the energy consumption will be a 26% higher than *E_min_*, if it is overestimated in 2 dB the energy consumption will be a 59% higher than *E_min_*, and if it is overestimated in 3 dB the energy consumption will be doubled with respect to *E_min_*. On the other hand, as shown in Figure 4(b), the number of received packets *n* will be lower than the desired *n_obj_* if the transmission power is underestimated. Notice that the behavior is quite sharp, especially for longer packets. For example, for 40-bytes packets, if the transmission power is underestimated in only 1.4 dB, the number of received packets will be halved with respect to the objective *n_obj_*, and if the transmission power is underestimated in more than 4 dB, the number of received packets will be practically zero.

The behavior shown in these figures is valid for both TOA-based and RSS-based localization schemes, but as the probability distribution of the distance estimation is different for the two localization schemes (see Appendix A and B), the probability distribution of ${\tilde{P}}_{\text{TX}}-P_{\text{TX}}^{*}$ will also be different. Therefore, the frequency of underestimations and overestimations of the transmission power will depend on the distance estimation method, and this will affect the probability distribution of the energy consumption and the number of received packets, as we will see next.

This error in the transmission power can be calculated as:

(17) $$P_{\text{TX}}^{\prime }-P_{\text{TX}}^{*}(dB)=10\eta \log\frac{\tilde{d}}{d}$$

where *d̃* is the estimated distance. Appendix A and B derive the expressions of *d̃* for TOA-based and RSS-based localization respectively. In a TOA-based localization scheme the distance estimation is given by Equation (24), therefore the difference between calculated and optimum transmission power will be given by:

(18) $$P_{\text{TX}}^{\prime }-P_{\text{TX}}^{*}=10\eta \log\frac{d+𝒩\;(0,\sigma )}{d}=10\eta \log 𝒩\;(1,\sigma /d)$$

where $\sigma =c\cdot \sigma_{t}/\sqrt{n}$ or $\sigma =c\cdot \sigma_{t}/2\sqrt{n}$, as explained in Appendix A. On the other hand, in a RSS-based localization scheme the distance estimation is given by Equation (28), thus the difference between calculated and optimum transmission power will be given by:

(19) $$P_{\text{TX}}^{\prime }-P_{\text{TX}}^{*}=𝒩\;(0,\sigma )$$

with $\sigma =\sigma_{\text{RSS}}/\sqrt{n}$.

**Figure 5.**
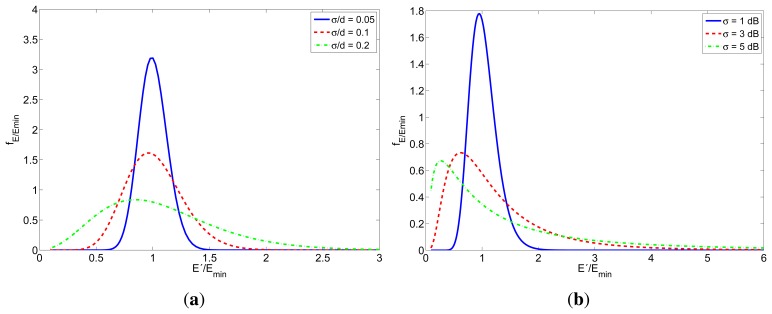
Probability density function of *E*′/*E_min_* for TOA-based and RSS-based localization schemes. (**a**) TOA; (**b**) RSS.

Figure 5 shows the probability density function of the relative energy consumption (*E*′/*E_min_*) for the cases of TOA-based and RSS-based localization. The different curves correspond to different values of *σ*/*d* and *σ* respectively. Notice that in the TOA case the individual values of *σ* and *d* do not have any influence, as long as the relation *σ*/*d* remains the same. This can be easily understand from Equation (18). In both cases, the probability of consuming more energy increases with *σ*. Furthermore, the mean value of *E*′/*E_min_* also increases when the standard deviation increases.

**Figure 6.**
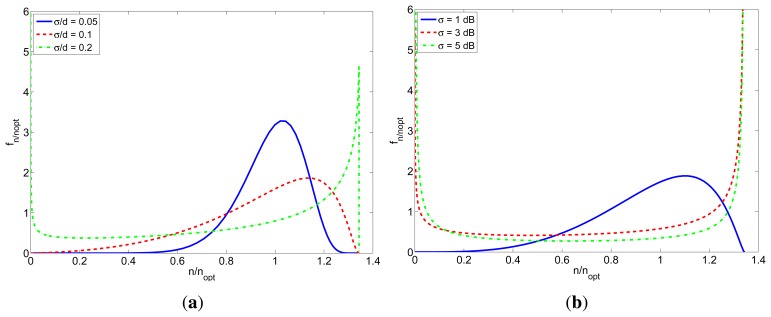
Probability density function of *n*/*n_obj_* for TOA-based and RSS-based localization schemes. (**a**) TOA; (**b**) RSS.

Figure 6 shows the probability density function of the relative number of received packets (*n*/*n_obj_*) for TOA-based and RSS-based localization. The different curves correspond to different values of *σ*/*d* and *σ* respectively, and were calculated for a packet length of *l* = 5 bytes (different packet lengths produce similar results, but a little more sharper as the packet length increases). In both cases, when the standard deviation is bigger, the probability of not receiving the desired number of packets is higher. It can be also seen that if the standard deviation is over a certain threshold, the pdf begins to be bimodal. This is because the margin of *SNR* for which the *PRP* takes intermediate values is small, so if the standard deviation is big enough, the probability of receiving packets with a very low or very high *SNR* (enough to make *PRP* = 0 or *PRP* = 1 respectively) is significant.

In conclusion, for both TOA and RSS-based localization methods, a greater variance in the measurements will produce a greater variance in the optimum transmission power estimation and thus, more energy consumption and a higher probability of losing packets.

### 4.4. Application to Real Hardware

The proposed strategy has been derived from the assumption that the power consumption of the transmitter is equal to the transmitted power. However, for real devices, this assumption may not be true due to the inefficiencies of the hardware. In this case, the theoretical strategy previously proposed has to be slightly modified, by substituting the transmission power *p_tx_* in Equation (5) by the power consumption at transmission *p_T_*, which in general may depend on the transmission power in a non-linear way. Therefore, the real energy consumption is related to the ideal one, given by Equation (5), by the following expression:

(20) $$E_{\text{real}}=E_{\text{TX}}\cdot \frac{p_{T}}{p_{\text{tx}}}=E_{\text{TX}}\cdot g(p_{\text{tx}})$$

where *g*(*p_tx_*) = *p_T_*/*p_tx_* is a nonlinear function of *p_tx_* that will be different for each type of hardware.

Similarly to the ideal case, we can minimize this energy consumption with respect to the *snr* by setting the derivative equal to zero (and working with the mean values of the random variables). After some algebraic calculations, we finally obtain the following expression:

(21) $$\begin{array}{ccc} {\frac{\partial E_{\text{real}}}{\partial \text{snr}}|}_{x=x^{*}}=0 & \Rightarrow & g(p_{\text{tx}})\cdot \left(2-\frac{8lx\cdot e^{-\frac{x^{2}}{2}}}{(1-Q(x))\cdot \sqrt{2\pi }}\right)+\frac{v_{b}}{W}\cdot x^{2}\cdot \frac{\partial g(p_{\text{tx}})}{\partial p_{\text{tx}}}\cdot \frac{d^{\eta }\;n_{\text{rx}}}{a}\cdot e^{\frac{1}{2}{\left(\frac{\sigma \cdot \ln10}{10}\right)}^{2}}=0 \end{array}$$

The value of *x* (and therefore, the value of *snr*) for which this expression is equal to 0 gives the minimum average energy consumption. Clearly, the result will depend on the hardware characteristics.

As an illustration, we next apply this method for two common radio chips used in wireless sensor networks devices: the ChipCon chips CC1000 and CC2420. Clearly, real devices also include other components apart from the radio chip, which will also affect the energy consumption. However, this example illustrates how to proceed in a general case where there is a nonlinear relationship between transmission power and consumption. Several values of *p_T_* as a function of *p_tx_* can be obtained from the datasheets of the two chips [30,31]. In order to have an approximate expression of *g*(*p_tx_*) and its derivative, we approximated these sets of points by an analytical expression of the form $g(p_{\text{tx}})=U\cdot p_{\text{tx}}^{V}$. Table 1 shows the values of *U* and *V* for the two chips and the correlation coefficient of the fitting. As it can be seen, there is a high correlation in both cases, which means that the considered expression fits well to the data.

Introducing this expression and its derivative in Equation (21), and after some algebraic manipulation, we finally obtain that the value of *x* for which the average energy consumption reaches a minimum (x*) satisfies the following expression:

(22) $$\frac{x^{*}e^{-x^{*2}/2}}{\left(1-Q(x^{*})\right)}=\frac{\sqrt{2\pi }}{4l}\cdot (1+V)$$

Thus, if we manage to achieve at the receiver the corresponding value of signal to noise ratio snr* (using Equation (8)), the real energy consumption will be minimized. Similarly to the ideal case, the optimum value of the *snr* at the receiver to achieve the lowest energy consumption does not depend on the accuracy we want to obtain or on the propagation characteristics, it only depends on the packet length *l* and on the *W*/*v_b_* ratio, *i.e.*, its value is constant for a given network and does not need to be calculated in real time. Again, as in the ideal case, there is an optimum value of *T* corresponding to snr*, which can be calculated from Equation (11). Finally, the corrections for the transmission power and the packet transmission rate can be calculated similarly to the ideal case.

In conclusion, the general receipt to apply the proposed method for a specific device is:

1. Characterize the energy consumption of the device as a function of the transmission power
2. Approximate this behavior with an analytical expression for *g*(*p_tx_*) = *p_T_*/*p_tx_*
3. Introduce this expression in Equation (21) and solve for *x*
4. Calculate the corresponding values of snr* (using Equation (8)) and T* (using Equation (11))
5. Calculate correction factors numerically as explained in Section 4.2

## 5. Performance Evaluation

In this section, we first describe a set of experiments that were carried out to characterize a real propagation environment in order to obtain realistic models to simulate the performance of the proposed strategy. Next, we include a numerical evaluation of the proposed transmission strategy that compares its performance with the performance of other transmission strategies in several scenarios.

### 5.1. Experimental Characterization of the Environment

In order to simulate a realistic situation, we carried out some experiments to characterize the propagation environment of our laboratory using Memsic's MICAz motes [32]. The objective was to check that the models proposed in Section 3 approximately reflect the behavior of our wireless network and, at the same time, to extract the values of some of the model parameters that we need for the simulations.

**Figure 7.**
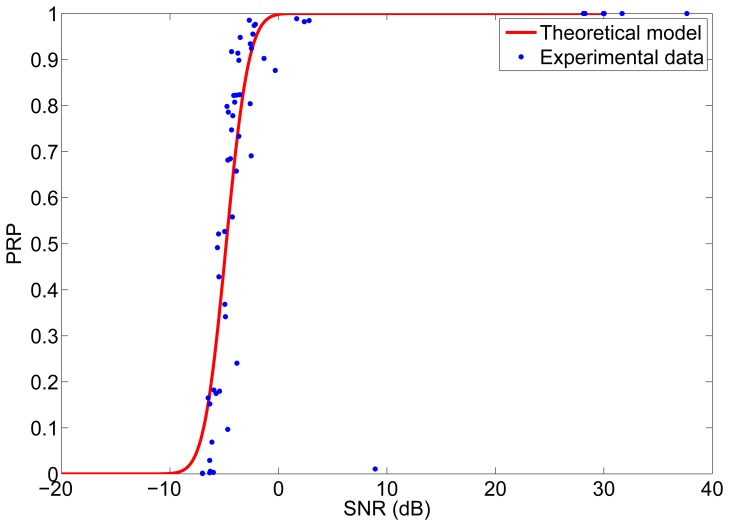
Relation between *PRP* and *SNR*.

The first set of experiments were carried out to determine the *PRP* as a function of the *snr*. These experiments were made with Memsic's MICAz motes. The MICAz is a 2.4 GHz mote with direct sequence spread spectrum radio, which uses an O-QPSK modulation scheme. Two motes were used for the experiments, one as transmitter and one as receiver. The experiments were carried out in a laboratory environment in which there were obstacles and people sitting or walking and interfering signals from other motes and other wireless systems working at the same frequency (WiFi). In each experiment, the transmitter node transmitted a packet of 8 bytes every second and the receiver node measured the RSS of the received packets, from which we obtained the *snr* at the receiver. The experiment was repeated for different distances between transmitter and receiver and for different transmission powers in order to have different values of *snr* at the receiver. The experimental results are shown in Figure 7, together with the theoretical model given by Equation (1). As it can be noticed in Figure 7, the experimental data adjust quite well to the model, with the exception of some outliers. From this experiment we obtained a value of *N_RX_* = −87 dBm for the noise level at the receiver.

**Figure 8.**
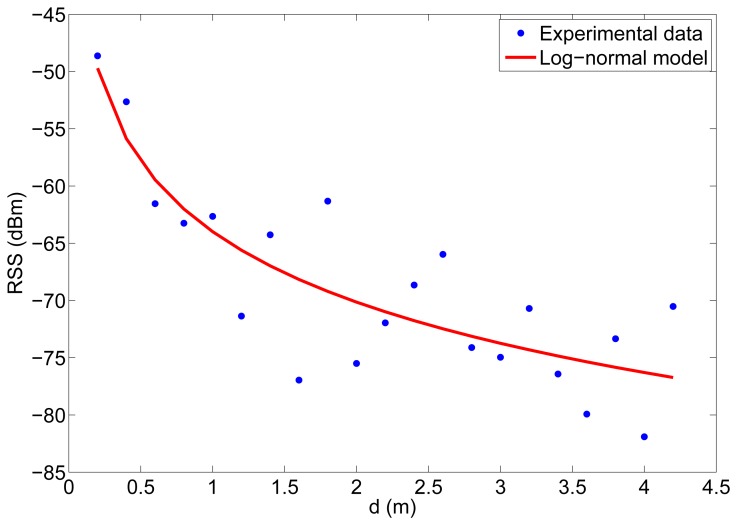
Experimental RSS measurements for different distances between two MICAz nodes. The log-normal channel model curve fitting is also represented.

We also made some experiments to characterize our radio channel, that is, to establish a relation between transmission and received power in terms of the distance. In these experiments, two MICAz motes were used again, with the same configuration as in the *PRP* experiments, and the tests were carried out in different rooms. The RSS of the received packets was measured for different distances between transmitter and receiver. At each position, several measurements of RSS were obtained, in order to average their values. In these experiments the transmitter used *P_TX_* = 0 dBm. The results from one of the experiments are shown in Figure 8. The curve in Figure 8 represents the fitting of the mean of Equation (2) to these experimental data and was calculated using the Levenberg–Marquardt algorithm, taking *d*_0_ = 1 m, *P_TX_* = 0 dBm and *η* and *A* as free parameters. From this experiment we obtained a value for the path loss exponent and the constant *A (η* = 2 and *A* = −64 dB). For other experiments, the estimated values of *A* were between −60 dB and −70 dB and the estimated values of *η* were between 2 and 3.

### 5.2. Simulation Results

We have run a set of simulations to evaluate the energy consumption of the proposed strategy in several scenarios. In these simulations, the performance of the proposed strategy is also compared with other two strategies: (1) sending *n* packets with the highest transmission power available by hardware and (2) sending *n* packets with a transmission power that depends on the estimated inter-node distance: *P_TX_*(*dBm*) = −*A* + 10*η* log*d̃*/*d*_0_ + *N_RX_*(*dBm*), which is the most common strategy when power adaptation is used (note that this is equivalent to using a transmission power such that the *SNR* at the receiver is 0 dB).

In these simulations, we considered networks composed of reference nodes and mobile nodes. In order to estimate the position of the mobile nodes, each reference node transmits packets to them. These nodes measure the RSS of the received packets and calculate their distance to each of the reference nodes. With these range estimations and the known positions of the reference nodes, the mobile nodes are able to calculate their own positions. As soon as they have an estimated value of their physical position, they can send packets to other nodes with the information about their estimated position and act themselves as reference nodes. Consequently, every node of the network will transmit packets to and receive packets from every other node within its communications range. Considering the transmission strategy proposed in the previous section for a single link, we applied it to the whole network by finding at each node the optimum transmission strategy $\left(P_{\text{TX}}^{*}\;\text{and}\;T^{*}\right)$ for its furthest neighbor (closer neighbors will be guaranteed to receive at least the same average number of packets). The transmission power *P_TX_* is adaptively modified, by estimating from Equation (3) the value necessary to achieve the desired SNR* at the furthest neighbor. As an estimate of the distance between the transmitter and its furthest neighbor is needed, the method is refined iteratively: first, the transmission power is set to an initial (high) value and a preliminary distance estimation is obtained; then, the transmission power is modified according to the distance estimation.

First, we simulated a network composed of four reference nodes deployed randomly in a 15 × 15 m^2^ room and one mobile node that stays in the center of the room during 20 seconds. The localization is done every second (*T*_0_ = 1 s) and the desired accuracy is such that *n* = 5. The communication channel was simulated using the log-normal model in Equation (2) with *A* = −65 dB, *η* = 2.5, *d*_0_ = 1 m and *σ* = 0 dB (ideal channel without shadowing). Packet reception was modeled with Equation (1), using *W* = 2 Mhz and *v_b_* = 250 kbps. The average energy consumption of 5000 simulations (50 different node placements, 100 simulations for each placement), which stays constant after the first localization interval, is shown in Table 2 for different values of the packet length. On the other hand, in this ideal simulation environment, the three strategies guarantee that the average number of received packets is *n* = 5 at the furthest node during the whole simulation. It can be seen that the proposed strategy gives the lowest energy consumption (about 47% the energy consumption of strategy #2 for *l* = 4) and, at the same time, guarantees that the average number of received packets is *n* = 5 at the furthest node. The length of the localization packets also has an influence in the energy consumption: the gain of the proposed method with respect to the other two is higher for lower values of *l*.

**Figure 9.**
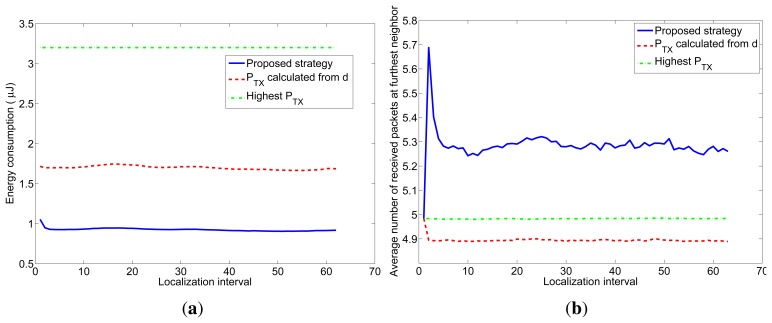
Average energy consumption and average number of received packets at the furthest node of the three strategies for each localization interval for an environment with shadowing.

Next, to simulate a more realistic scenario, we added shadowing with *σ* = 2 dB and node movement to the previous simulation. We simulated a 7m-radius circular trajectory centered in the simulated area. The influence of the shadowing is twofold, as explained in Section 4.2. On one hand, the distances are usually underestimated, so the transmission power calculated from them to reach the receiver with the desired *snr* has to be corrected with the correction margin given at Figure 2. On the other hand, even when the optimum transmission power is used, the final *snr* at the receiver may not be the desired one due to the effects of shadowing. To counteract this effect, we have corrected the number of transmitted packets with the factor given at Figure 3. The average results of 5000 simulations are shown in Figure 9. Figure 9(a) shows the average energy consumption of the three strategies for each localization interval and Figure 9(b) shows the average number of received packets at the furthest node. It can be seen that the proposed strategy gives the lowest energy consumption (about 54% the energy consumption of strategy #2) and, at the same time, guarantees that, at least, the average number of received packets at the furthest node is *n* = 5. These results were obtained for localization packets of length *l* = 4 bytes. Table 3 shows the energy consumption of the proposed strategy for other values of *l* and Table 4 shows the average number of received packets at the furthest neighbor. It can be seen that the length of the localization packets has an effect on the performance of the proposed strategy: the longer the packet is, the lower is the gain in energy consumption with respect to other strategies. This is because when *l* increases, the optimum *snr* increases as well and gets closer to 1 (*SNR* = 0 dB), so the difference in energy consumption with respect to strategy #2 decreases. In any case, the proposed strategy always assures a given number of received packets, in contrast to strategy #2 and, moreover, entails energy savings with respect to other strategies.

**Figure 10.**
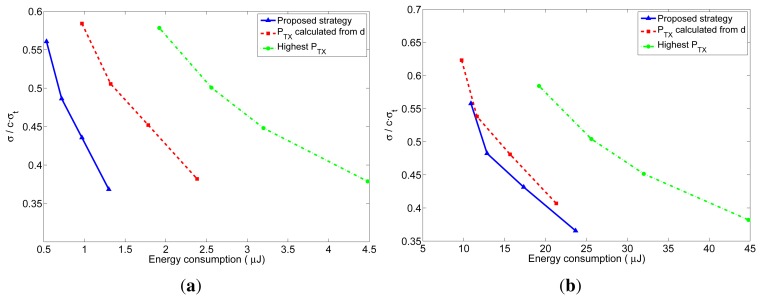
Relation between the average energy consumption per localization and the TOA-based distance estimation accuracy for the three strategies. (**a**) l = 4 bytes; (**b**) l = 40 bytes.

**Figure 11.**
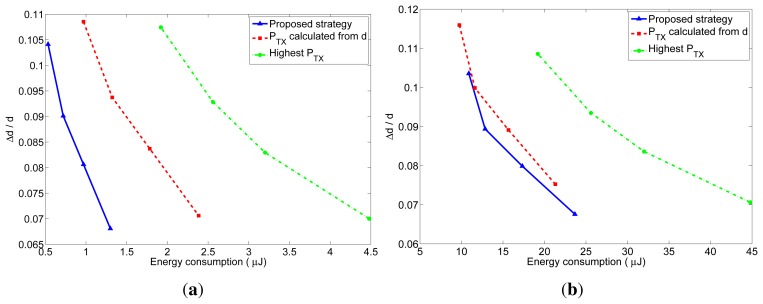
Relation between the average energy consumption per localization and the RSS-based distance estimation accuracy for the three strategies. (**a**) l = 4 bytes; (**b**) l = 40 bytes.

As explained before, the number of received packets at the furthest neighbor determines the accuracy of the distance estimation at these nodes. The accuracy of the distance estimations in closer neighbors will be at least as good as the one at the furthest neighbor. In order to better understand the behavior of the proposed transmission strategy, we have represented in Figures 10 and 11 the relation between the average energy consumption and the accuracy of the distance estimations for TOA and RSS measurements respectively, obtained from the previous realistic simulations for different values of *n*. The x-axis represents the average energy consumption of the whole network at each localization interval, which was calculated by summing up the energy consumption of all the nodes at each localization interval, and averaging the result over time (excluding the first localization intervals, in order to eliminate the initialization effects) and over the 5000 simulations. The y-axis represents the distance estimation accuracy at the furthest neighbor, which was calculated from the average number of received packets at the furthest neighbors. This value was obtained by taking, at each localization interval, the furthest neighbor for each node of the network, and averaging the number of packets received by these nodes. Then the results were averaged over time (excluding the first localization intervals) and over the 5000 simulations. From the number of received packets, the accuracy of the distance estimation was calculated according to the expressions of the TOA-based and RSS-based distance estimations which are derived in Appendix A and B. In the TOA case, the estimated distance is a Gaussian random variable given by Equation (24) with mean equal to the real distance and standard deviation given by Equation (25) for one-way TOA measurements or by Equation (26) for two-way TOA measurements. Figure 10 represents the accuracy as the ratio between the standard deviation of the distance estimation (*σ*) and the factor *c* · *σ_t_*, which is the standard deviation corresponding to one one-way TOA measurement. Therefore, the accuracy in this figure should be read as relative to the accuracy of one TOA measurement. In the RSS case, the estimated distance is a log-normal random variable with variance given by Equation (32). Figure 11 represents the accuracy as the ratio between the standard deviation of the distance estimation (Δ*d*) and the real distance *d*, where the distance estimation variance was calculated for a channel with *σ_RSS_* = 2 dB (for other values of *σ_RSS_* the particular values of the relative accuracy change: they get bigger for bigger values of *σ_RSS_*, but the relation between the three strategies remains the same). Therefore, the accuracy in this figure should be read as a relative error in the distance (a percentage of the real distance). From these figures, it can be clearly noticed that the proposed method has a better performance regarding both the energy consumption and the accuracy of the distance estimations. The gain with respect to the other strategies is especially noticeable for short localization packets, which is the usual situation, as these packets do not carry any data information.

**Figure 12.**
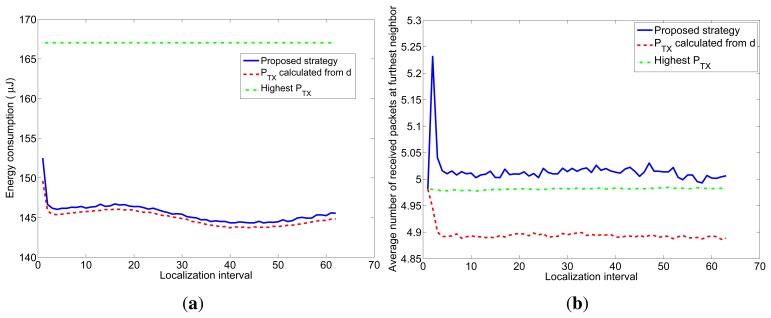
Average energy consumption and average number of received packets at the furthest node of the three strategies for the CC2420 consumption model.

Finally, we have evaluated the performance of the proposed strategy for a case in which the power consumption is not equal to the transmitted power (which is usually the case for real devices). To this end, we repeated the previous simulation experiments considering the energy model for the CC2420 radio chip described in Section 4.4 and following the steps described in that section to adjust the proposed strategy. Figure 12 shows the results for *n* = 5 and *l* = 4 bytes.

**Figure 13.**
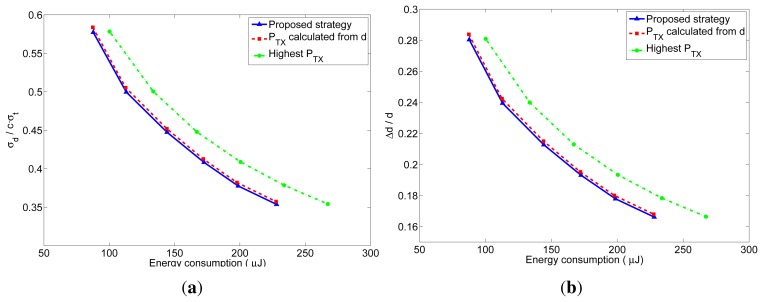
Relation between the average energy consumption per localization and the distance estimation accuracy for the three strategies for *l* = 4 bytes. (**a**) TOA-based localization; (**b**) RSS-based localization.

It can be seen in Figure 12(b) that the proposed strategy is again the only one that guarantees the desired accuracy (*n* = 5), but in this case its energy consumption (Figure 12(a)) is a little bit higher than for strategy #2 (which does not guarantee the desired accuracy). To better understand this behavior, we show in Figure 13 the relation between the average energy consumption and the accuracy of the distance estimations for the different strategies.

As it can be noticed, for a given energy consumption, the proposed strategy achieves better accuracy than strategy #2, and for a given accuracy, the proposed strategy has lower energy consumption. Our proposed method shows still a gain, but of lower entity with respect to the ideal case shown in Figures 10 and 11. The reason is that for this particular hardware, the optimum value of the *SNR* is closer to 0 dBm, thus, there is not a big margin for improvements, as strategy #2 is practically a strategy that tries to achieve a *SNR* = 0 dBm at the receiver.

To sum up, the proposed strategy always guarantees a desired accuracy in the distance estimation and, at the same time, yields energy savings, especially when the optimum value of the *SNR* is low. As this optimum value depends on the particular energy consumption characteristics of the wireless device, bigger savings are expected for devices with more similar characteristics to that of the ideal case.

## 6. Conclusions and Further Work

In this paper, we have presented an analysis of the energy consumption during the localization process of a wireless sensor network. We have optimized the transmission strategy (in terms of transmission power and packet transmission rate) at each node with the aim of obtaining certain accuracy in the RSS or TOA measurements (expressed by the number of packets that the receiver node should average) while minimizing the energy consumption in the network. We have shown that the optimum transmission strategy consists in sending packets with such a transmission power $P_{\text{TX}}^{*}$ that the *snr* at the receiver is the optimum and a packet rate of 1/T*, where T* depends on the desired accuracy and the optimum value of the *snr*. However, we have also seen that the value of $P_{\text{TX}}^{*}$ depends on the distance between nodes, which is not known in advance, as it is the parameter that is estimated during localization. In order to apply the results above, the transmission power has to be estimated iteratively.

We have also proposed some modifications to the theoretical optimal strategy in order to correct the effects of the propagation channel when applying this strategy in a real scenario. In particular, we have proposed how to correct these effects in the case of RSS-based localization, by adding a correction margin to the estimated transmission power and correcting the number of transmitted packets by a factor that depends on the packet length and the value of *W*/*v_b_*. The proposed strategy with its corrections was proved to guarantee a desired number of received packets at the receiver (necessary to obtain a given accuracy in the distance estimations) while having at the same time lower energy consumption than other strategies.

In further work we are planning to evaluate the performance of the proposed strategy using real-field deployments. In particular, we are implementing the embedded versions of the strategy in MICAz motes (under TinyOS 2.1), to experimentally measure and evaluate the energy consumption during the localization when the proposed strategy is executed in these resource-constrained devices.

Future work on studying the effects that the accuracy of the radio measurement has on the distance estimation for TOA-based and RSS-based localization techniques will allow extracting some guidelines for the calculation of the required average number of received packets, depending on the localization accuracy requirements and on the geometric configuration of the network. Further work would also include the analysis of other channel and receiver models and the evaluation of variant channels. Furthermore, the value of the localization period *T*_0_, which we have considered as constant, may change in order to adapt to the mobility characteristics of the mobile node. The combination of the proposed optimization with the optimization of the localization period may lead to further energy savings and could be a promising line of future work.

## Figures and Tables

**Table 1.** Fitting of $U\cdot p_{\text{tx}}^{V}$ to the real values of *g*(*p_tx_*) = *p_T_*/*p_tx_* for the CC1000 and CC2420 radio chips.

	**CC1000**	**CC2420**
*U*	36.8596	48.8470
*V*	−0.8256	−0.8728
*R*^2^	0.9945	0.9986

**Table 2.** Average energy consumption (in *μ* J) of the three strategies for different packet lengths.

	***l* = 4**	***l* = 10**	***l* = 20**	***l* = 40**
Highest *P_TX_*	3.20	8.00	16.00	32.00
*P_TX_* calculated from *d*	1.26	2.98	5.87	11.58
Proposed strategy	0.60	1.79	4.24	9.19

**Table 3.** Energy consumption of the proposed strategy expressed in % with respect to strategy #2.

***l* = 4**	***l* = 10**	***l* = 20**	***l* = 40**
54%	73%	92%	110%

**Table 4.** Average number of received packets at the furthest neighbor.

	***l* = 4**	***l* = 10**	***l* = 20**	***l* = 40**
Highest *P_TX_*	4.98	4.96	4.93	4.90
*P_TX_* calculated from *d*	4.89	4.76	4.58	4.32
Proposed strategy	5.28	5.31	5.33	5.37

## Appendix

### A. Distance Estimation Error in TOA-Based Localization

We assume that the measurement of the time *t* required for the signal to travel between two nodes (one-way or round trip) is affected by an additive Gaussian random noise with variance $\sigma_{t}^{2}$. The variance of the TOA measurement can be reduced if *n* measurements are averaged, as mentioned in Section 3. Specifically, the averaged measured value of the time *t* can be expressed as:

(23) $${\tilde{t}}_{av}=t+𝒩\;\left(0,\frac{\sigma_{t}}{\sqrt{n}}\right)$$

On the other hand, the distance *d* between transmitter and receiver is easily related to the time *t: d* = *c*·*t* for one-way TOA measurements or *d* = *c*·*t*/2 for two-way TOA measurements, where *c* is the speed of light. Thus, the estimated distance *d̃* will be a Gaussian random variable:

(24) $$\tilde{d}=d+𝒩\;(0,\sigma )$$

where the value of *σ* depends on the measurement type:

For one-way TOA measurements:

(25) $$\sigma =c\cdot \sigma_{t}/\sqrt{n}$$

For two-way TOA measurements:

(26) $$\sigma =c\cdot \sigma_{t}/2\sqrt{n}$$

It can be noticed that the error (or variance) in the distance estimation does not depend on the actual distance between transmitter and receiver. From these expressions, we can obtain the number of packets that we need to receive in order to achieve a given accuracy in the distance estimation.

### B. Distance Estimation Error in RSS-Based Localization

Assuming that the measurement of the received signal strength *P_RX_* is affected by an additive Gaussian random noise with variance $\sigma_{\text{RSS}}^{2}$, the variance of the RSS measurement can be reduced if *n* measurements are averaged. In this case, the averaged measured value of the RSS can be expressed as:

(27) $${\tilde{P}}_{\text{RX}av}=P_{\text{RX}}+𝒩\;\left(0,\frac{\sigma_{\text{RSS}}}{\sqrt{n}}\right)$$

Note that this variance $\sigma_{\text{RSS}}^{2}$ coincides with the variance of the log-normal shadowing *σ*^2^ if the environment is dynamic or the nodes are moving, whereas in a completely static situation, it will be lower than *σ*^2^, as explained in Section 3.2.

On the other hand, the distance *d* between transmitter and receiver is related to the received signal strength through the channel model. Assuming the log-normal channel model (see Section 3.2) as valid we have that the estimated distance *d̃* will be a random variable defined by:

(28) $$\tilde{d}=d\cdot 10^{\frac{𝒩\;(0,\sigma_{\text{RSS}}/\sqrt{n})}{10\eta }}$$

In order to calculate the variance of this distance estimation, *d̃* can be expressed as a function of a log-normal random variable:

(29) $$\tilde{d}=d\cdot 10^{\frac{𝒩\;(0,\sigma_{\text{RSS}}/\sqrt{n})}{10\eta }}=d\cdot 10^{𝒩\;\left(0,\frac{\sigma_{\text{RSS}}}{10\eta \sqrt{n}}\right)}=d\cdot X$$

where *X* is a log-normal random variable with parameters *μ_x_* = 0 and $\sigma_{x}=\sigma_{\text{RSS}}\cdot \ln10/\left(10\eta \sqrt{n}\right)$, that is, ln *X* is a zero-mean normal distribution with standard deviation *σ_x_*:

(30) $$\log X=𝒩\;\left(0,\frac{\sigma_{\text{RSS}}}{10\eta \sqrt{n}}\right)$$

(31) $$\ln X=\ln10\cdot 𝒩\;\left(0,\frac{\sigma_{\text{RSS}}}{10\eta \sqrt{n}}\right)=𝒩\;\left(0,\frac{\sigma_{\text{RSS}}\cdot \ln10}{10\eta \sqrt{n}}\right)$$

The variance of a log-normal distribution with parameters *μ_x_* and *σ_x_* is $e^{2\mu_{x}+\sigma_{x}^{2}}\cdot \left(e^{\sigma_{x}^{2}}-1\right)$ [33]. Thus, the variance of the distance estimation *d̃* is given by:

(32) $$\text{V ar}(\tilde{d})=d^{2}\cdot \text{V ar}(X)=d^{2}\cdot (e^{\alpha }\cdot (e^{\alpha }-1))$$

with

(33) $$\alpha =\sigma_{x}^{2}={\left(\frac{\sigma_{\text{RSS}}\cdot \ln10}{10\eta \sqrt{n}}\right)}^{2}$$

It can be noticed that, in this case, the error in the distance estimation does depend on the actual distance between transmitter and receiver. The greater the distance is, the greater the variance of the distance estimator becomes. From this expression, we can obtain the number of packets that we need to receive in order to achieve a given accuracy in the distance estimation.
